# Fibroblasts to Keratinocytes Redox Signaling: The Possible Role of ROS in Psoriatic Plaque Formation

**DOI:** 10.3390/antiox8110566

**Published:** 2019-11-18

**Authors:** Victoria Barygina, Matteo Becatti, Francesca Prignano, Torello Lotti, Niccolò Taddei, Claudia Fiorillo

**Affiliations:** 1 Department of Biomedical, Experimental and Clinical Sciences “Mario Serio”, University of Florence, viale Morgagni 50, 50134 Florence, Italy; matteo.becatti@unifi.it (M.B.); niccolo.taddei@unifi.it (N.T.); claudia.fiorillo@unifi.it (C.F.); 2 Department of Dermatological Sciences, University of Florence, Villa Basilewsky, via Lorenzo Il Magnifico 104, 50129 Florence, Italy; francesca.prignano@unifi.it; 3 Department of Nuclear, Subnuclear and Radiation Physics, Via Plinio 44, 00193 Rome, Italy; professor@torellolotti.it

**Keywords:** NOX4, psoriasis, ROS signalling, fibroblasts, keratinocytes, co-culture, redox, NADPH oxidase

## Abstract

Although the role of reactive oxygen species-mediated (ROS-mediated) signalling in physiologic and pathologic skin conditions has been proven, no data exist on the skin cells ROS-mediated communication. Primary fibroblasts were obtained from lesional and non-lesional skin of psoriatic patients. ROS, superoxide anion, calcium and nitric oxide levels and lipoperoxidation markers and total antioxidant content were measured in fibroblasts. NADPH oxidase activity and NOX1, 2 and 4 expressions were assayed and NOX4 silencing was performed. Fibroblasts and healthy keratinocytes co-culture was performed. MAPK pathways activation was studied in fibroblasts and in co-cultured healthy keratinocytes. Increased intracellular calcium, •NO and ROS levels as well as an enhanced NADPH oxidase 4 (NOX4)–mediated extracellular ROS release was shown in lesional psoriatic vs. control fibroblasts. Upon co-culture with lesional fibroblasts, keratinocytes showed p38 and ERK MAPKs pathways activation, ROS, Ca^2+^ and •NO increase and cell cycle acceleration. Notably, NOX4 knockdown significantly reduced the observed effects of lesional fibroblasts on keratinocyte cell cycle progression. Co-culture with non-lesional psoriatic and control fibroblasts induced slight cell cycle acceleration, but notable intracellular ROS accumulation and ERK MAPK activation in keratinocytes. Collectively, our data demonstrate that NOX4 expressed in dermal fibroblasts is essential for the redox paracrine regulation of epidermal keratinocytes proliferation.

## 1. Introduction

Reactive oxygen species (ROS) are messenger molecules that play a prominent role in cell metabolism-regulating cell proliferation, differentiation and death [1]. Among them, hydrogen peroxide (H_2_O_2_) is one of the most intriguing ROS. It reacts slowly with most molecules, however, possesses a high affinity to cysteine residues surrounded by particular sequences of amino acids. This explains the selectivity of H_2_O_2_ for thiolates moieties in active sites of various enzymes [2]. H_2_O_2_ is produced by spontaneous and enzyme-mediated dismutation of superoxide (O_2_•^−^) and is mostly formed in the sites of O_2_•^−^ production. Two well-known sources of O_2_•^−^ in the living cell are the mitochondria electron transport chain (ETC) and the nicotinamide adenine dinucleotide 2′-phosphate (NADPH) oxidases. O_2_•^−^ represents a side-product of ETC activity where it forms due to electron “escape” and reduction of dioxygen (O_2_) present at high concentration in the mitochondrial membrane [3]. O_2_•^−^ formation in mitochondria is a hallmark of mitochondrial activity [4]. In contrast with ETC, NADPH oxidases produce ROS as their main product. Seven members of NADPH oxidase family are actually described: NOX1-5, Duox1 and Duox2. All seven isoforms are flavocytochromes anchored to the membrane through six transmembrane helices binding two haem cofactors (N-terminal domain). The C-terminal dehydrogenase domain binds flavin adenine dinucleotide (FAD) and NADPH and allows electron transfer to the haem and further across the membrane to molecular oxygen [5]. Interestingly, while NOX1, NOX2 and NOX3 are known to produce O_2_•^−^, the other three members of the NADPH oxidase family produce H_2_O_2_ as the main product. For instance, NOX4 produces 90% of H_2_O_2_ and 10% of O_2_•^−^ by the currently accepted ‘two sequential single-electron reductions’ mechanism [5]. 

NADPH oxidases have been extensively studied due to their involvement in a number of human pathologies, one of which is psoriasis vulgaris. Psoriasis is a dermatologic condition characterized by hyper-proliferation and altered keratinocytes differentiation that leads to the formation of psoriatic plaques—red scaly patches on the skin surface. In the sites of psoriatic lesions, the skin architecture is altered and characterized by the formation of pronounced epidermal protrusions into dermis greatly increasing the area of contact between epidermis and dermis. These morphological changes are associated with the notable redox imbalance. Our previous studies showed an increased intracellular ROS production and an enhanced NADPH oxidase activity in fibroblasts obtained from lesional skin of psoriatic patients [6]. This data is in line with the findings of other authors who showed O_2_•^−^ production, mitochondrial superoxide dismutase activity (Mn-SOD) and protein carbonylation level [7,8] to be significantly increased in fibroblasts obtained from involved and uninvolved skin of psoriatic patients in comparison to healthy skin fibroblasts. 

In the present study, an in-depth investigation on redox status in fibroblasts obtained from lesional (“LES”, the area of psoriatic plaque) and non-lesional (“nLES”, visibly unaffected) skin of psoriatic patients with respect to primary control (CTR) fibroblasts obtained from healthy donors was performed. In particular, oxidative stress markers and NADPH oxidase activity in nLES and LES fibroblasts were investigated. Furthermore, in order to identify possible ROS sources in psoriatic fibroblasts, three members of the NADPH oxidase family were studied. Co-culture experiments of fibroblasts with healthy primary human keratinocytes were also performed in order to assess the possible effects of fibroblast-derived ROS on the proliferation rate and redox balance of keratinocytes. Finally, the involvement of stress-sensitive molecular pathways was investigated in psoriatic versus CTR fibroblasts and in keratinocytes co-cultured with the fibroblasts.

## 2. Materials and Methods

### 2.1. Reagents and Antibodies

The information of all reagents and antibodies used in the study is summarized in Table 1 and Table 2.

### 2.2. Patients

Five patients affected by plaque psoriasis and four healthy controls matched by age, and body mass index (BMI) were enrolled in this study approved by the Ethics Committee of Azienda Ospedaliero-Universitaria Careggi (N10709_bio from 27.03.2017) and conducted according to the Helsinki declaration. The demographic and clinical information for each patient and healthy subject is summarized in Table 3. The patients did not comply with the pruritus at the site of the lesions chosen for the biopsy. No subjects involved in the study followed any systemic therapy before the study or had a history of any disease, e.g., diabetes mellitus and atherosclerosis, which might affect the redox status.

### 2.3. Primary Fibroblasts and Keratinocytes Isolation and Setting Up of Cell Cultures

Fibroblasts isolation was performed as previously reported [6,9,10,11]. Briefly, 5 mm punch-biopsies were obtained from LES and nLES skin of psoriatic patients and from the skin of healthy controls (CTR). With nLES skin, we intend to use the perilesional symptom-free area of the skin. Immediately after, biopsies were subjected to the procedure of epidermal-dermal separation in 0.5% dispase II solution in DMEM containing antibiotics and antimycotics overnight at 4 °C. As follows, epidermis and dermis were easily detached with the forceps and washed with ice-cold PBS. In order to establish the primary fibroblasts, dermis was cut in small pieces, dried for 5–10 min on-air and covered by the cover-glass performing a moderate pressure. A complete DMEM (20% FBS, 1 g/L glucose, 100 units/mL of penicillin, 100 µg/mL of streptomycin and 0,25 µg/mL of Amphotericin B) was added to the dish and changed every two days. After 10–12 days the semi-confluent layer of fibroblasts was observed in the dish. At this point, the dermal pieces were transferred to a new dish where the process of fibroblasts migration continued. The semi-confluent fibroblasts were trypsinized and cultivated in DMEM (10% FBS, 1 g/L glucose, 100 units/mL of penicillin, 100 µg/mL of streptomycin and 0,25 µg/mL of Amphotericin B) at 37 °C. The cells were frozen or used in experiments at their passages 2 to 5.

In order to establish the primary keratinocyte culture from control skin, the epidermis was chopped with a sterile scalpel into fine segments immediately after the separation from the dermis. Afterward, the epidermal slices were incubated in 0.25% Trypsin-EDTA solution (porcine trypsin) at 37 °C with frequent pipetting until the epidermal slice started to disaggregate into single cells. At this point, the pre-heated PBS was added to the dish and the cells were filtered through a cell strainer filter with 70 μm pores to obtain a single-cell suspension, which was further centrifuged for 5 min at 1000 rpm, resuspended in PBS and centrifuged again. The pellet was resuspended in KGM medium completed with all SingleQuotsTM Supplements and Growth Factors and 1% antibiotic-antimycotic solution. The anti-vimentin and anti-pan-cytokeratin immunocytochemical staining were further performed for both fibroblasts and keratinocytes cell cultures in order to verify the cell type purity. 

Prior to studies, the cell cultures were tested for Mycoplasma infection by DAPI staining and confocal microscope imaging. Moreover, when the primary cultures were used in laboratory, the surfaces of the cell incubator (every week) and cell culture hood (every day) were disinfected with anti-Mycoplasma-EX Spray Surface Disinfectant (PromoCell GmbH, Heidelberg, Germany). All cell cultures used in the present study were negative for Mycoplasma infection.

### 2.4. Contactless Keratinocytes-Fibroblasts Co-Incubation Protocol

**Figure 1:**
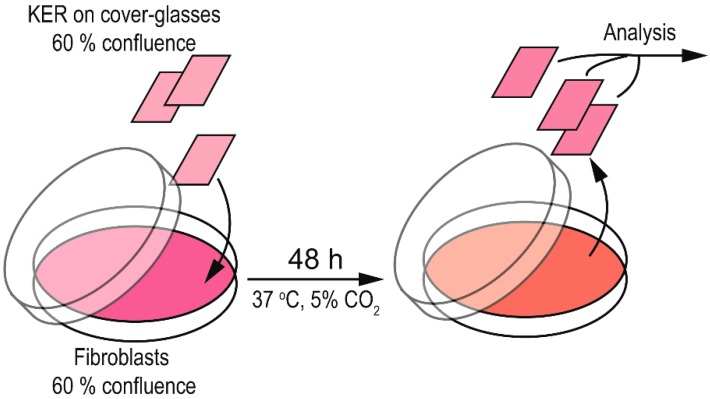
Contactless keratinocytes-fibroblasts co-incubation protocol. Keratinocytes (KER) were grown on cover-glasses (1.5 cm^2^) until semi-confluence (generally for 24 h) and put in Petri dish (diameter 10 cm) with semi-confluent fibroblasts. After 48 h of co-incubation, the KER–containing cover-glasses were removed from the Petri dishes, KER were trypsinized and examined.

In order to study the effect of ROS-hyper-producing fibroblasts on redox homeostasis of primary keratinocytes (KER) obtained from healthy subjects, KER were co-cultured with CTR, nLES and LES following the contactless protocol (Figure 1). Briefly, keratinocytes were plated on cover glasses, allowed to grow for 24 h and further put in Petri dish with semi-confluent fibroblasts seeded 24 h before. In this condition, continuous conditioning of KER by the fibroblasts factors is allowed although the two cell types are physically separated. After 24 h of co-incubation, the medium was changed. After 48 h of co-incubation, the KER-cover glasses were extracted from the Petri dishes and the keratinocytes were trypsinized and prepared for further studies. 

### 2.5. Indirect Immunocytochemistry (ICC)

Cells were fixed with 3% paraformaldehyde in PBS for 20 min at room temperature and treated with 0.5% Triton X-100 for 10 min on ice. The cells were thoroughly washed with PBS and incubated with primary antibodies (Table 2) diluted in PBS for 1 h at room temperature in a humid chamber in the dark. After washing with PBS, the cells were incubated with secondary antibodies (Table 2) diluted in PBS for 45 min at room temperature, dehydrated in alcohol gradient and embedded in Moviol containing DABCO (1,4-diazobicyclo[2,2,2]octane) (Sigma-Aldrich Italy S.r.l., Milan, Italy). The preparations were studied with Leica TCS SP5 Confocal Microscope (Mannheim, Germany) using 63×/NA 1.25 Plan Neofluar objectives (0.5 μm optical slice thickness, z-sections collected at 0.3 μm intervals). The excitation and emission wavelengths were 488 nm and 495–550 nm, respectively. If not stated alternatively the ICC protocol was the same for all experiments. The ICC staining was performed at the passages 0 and 5 for all psoriatic and control fibroblasts and for control keratinocytes.

### 2.6. Cell Cycle Progression Analysis

Cells were trypsinized, washed with PBS, resuspended in 0.5 mL of PBS containing 20 mg/mL propidium Iodide and left in dark at +4 °C for 1 h. Cell cycle distribution was analyzed by flow cytometry (FACSCanto, Becton-Dickinson, San Jose, CA, USA) and by FlowJo software (free software, Becton-Dickinson, San Jose, CA, USA). The percentage of cells in senescence/Gap 1 (G0/G1), synthesis (S) and Gap 2/mitotic (G2/M) phases were determined for the control and experimental conditions and averaged across triplicate experiments.

### 2.7. Assessment of Oxidative Stress Markers in Living Fibroblasts by Confocal Microscopy

CTR, nLES and LES fibroblasts were grown in glass-bottom Petri dishes (Nunc, Roskilde, Denmark) for confocal studies. At semi-confluence the DMEM was replaced with pre-heated serum-free and phenol red-free RPMI medium containing fluorescent probes H_2_DCF-DA (2.5 µM), DAF-FM (2.5 µM), Fluo-3 (2.5 µM), or MitoSOX (3 µM) dissolved in 0.1% DMSO and Pluronic acid F-127 (0.01% *w/v*) in order to visualize the intracellular ROS, nitric oxide (•NO), Ca^2+^ or mitochondrial O_2_•^−^ production levels, respectively. After 30 min incubation at 37 °C and three wash cycles with pre-heated PBS the RPMI medium was added and the cells were incubated at 37 °C for another 30 min prior to the confocal imaging. The fluorescence was detected in living cells at excitation and emission wavelengths of 488 and 520 nm, respectively, using a Leica TCS SP5 Confocal Microscope (Mannheim, Germany) equipped with an argon laser source. The observations were performed by collecting the emitted fluorescence with a Leica 40× oil immersion objective. 

### 2.8. Assessment of Intracellular Oxidative Stress Markers and Intracellular Ca^2+^ Levels in Living Fibroblasts and Keratinocytes by FACS Analysis

To determine intracellular ROS, •NO, Ca^2+^ and mitochondrial O_2_•^−^ production, fibroblasts/keratinocytes were trypsinized, washed with PBS and resuspended in serum- and phenol red-free RPMI medium containing 1 μM of the following fluorescent probes: H_2_DCF-DA, DAF- FM DA, Fluo-3 and MitoSOX, respectively. After 30 min of incubation in the dark at 37 °C, cells were washed three times, resuspended in PBS and fluorescence was analyzed by FACSCanto flow cytometer (Becton-Dickinson, San Jose, CA, USA).

### 2.9. Whole Cell Homogenates Preparation

After trypsinization, fibroblasts (1 × 10^6^) were resuspended on ice in 150 µL of lysis buffer (20 mM Tris-HCl pH8, 1% Triton X-100, 10% (*v/v*) glycerol, 137 mM NaCl, 2 mM EDTA and 6 M urea supplemented with 0.2 mM PMSF, 10 mg/mL leupeptin + aprotinin). Samples were then subjected to three freeze–thaw cycles and sonicated three times (5 s each) on ice, centrifuged at 14,000× *g* for 10 min at 4 °C, and the supernatant was collected. Protein concentration was determined according to the Bradford method [12]. Samples were stored at –80 °C before use. 

### 2.10. Thiobarbituric Acid Reactive Substance (TBARS) Evaluation

Malondialdehyde (MDA) is the final product of fatty acid peroxidation. MDA levels in CTR, nLES and LES fibroblasts homogenates were quantified using TBARS assay kit (Oxitek-ZeptoMetrix Corporation Buffalo, NY, USA) following the manufacturer protocol. An amount of 5 µL of whole-cell homogenates was used per sample. The fluorescence emission of the recovered supernatant was measured with an excitation wavelength of 530 nm and an emission wavelength of 550 nm, using a Perkin-Elmer LS55 spectrofluorimeter (Waltham, MA, USA).

### 2.11. Oxygen Radical Antioxidant Capacity (ORAC) Assay

ORAC assay was performed on homogenates of CTR, nLES and LES fibroblasts. The reaction was carried out in 96-well black microplates (Nunc, Roskilde, Denmark) and Trolox (10–200 μM) was used as standard. The amount of sample per well was calculated in consideration of 4 μg of protein/well. Final assay mixture of the total volume (200 μL) contained: 70 μL of sample diluted in 75 mM phosphate buffer (pH 7) and 100 μL of reagent fluorescein at 6 nM final concentration. After 10 min of incubation in the dark at 37 °C, 30 μL of pre-heated at 37 °C AAPH solution (final concentration of AAPH—200 mM) were added to each well using multiwall channel pipette. The fluorescence was recorded using a fluorometric microplate reader Fluoroskan Ascent (Thermo Electron Corp., Vantaa, Finland) at 5 min intervals for 2 h at excitation and emission wavelengths of 485 and 537 nm, respectively. All assays were conducted in triplicates and at least two independent tests were done for each sample. The area under curve (AUC) was calculated for each sample by integrating the relative fluorescence curve. Regression equations obtained from the net value of Trolox was used to calculate the ORAC value for each assay. Final ORAC values were expressed as μmol of Trolox equivalent (TE) per mg of protein (μmol TE/mg).

### 2.12. NADPH Oxidase Activity by Luminometric Assay

In order to measure the extracellular ROS production by CTR, nLES and LES fibroblasts, the cells were trypsinized, washed with PBS and 3 × 10^5^ of cells per sample were resuspended in 125 μL of Krebs-HEPES buffer (99 mM NaCl, 4.7 mM KCl, 1.2 mM MgSO_4_, 1 mM KH_2_PO_4_, 1.9 mM CaCl_2_, 25 mM NaHCO_3_, 20 mM HEPES, and 11.1 mM glucose pH 7.44) and placed in tubes for luminometric assay. After 5 min of incubation at 37 °C, lucigenin (25 μM) was added to the sample. As soon as luminescence level stabilized (in 10 min), the blank value of luminescence was registered by Lumat LB 9507 single-tube luminometer (Berthold Technologies GmbH & Co. KG, Bad Wildbad, Germany). NADPH was then added to the sample at the final concentration of 500 μM and luminescence was registered with 1 min interval for 20 min. Between readings, the cells were maintained at 37 °C. NADPH-stimulated extracellular ROS production was completely abated in fibroblasts pre-incubated for 30 min with the flavoenzyme inhibitor diphenyleneiodonium chloride (DPI, 20 μM) confirming that NADPH oxidase is the source of extracellular ROS production in living fibroblasts. When the curve of NADPH oxidase activity reached the plateau, the SOD (final concentration 450 U/mL) was added to the sample to monitor the O_2_•^−^ to H_2_O_2_ extracellular production ratio. NADPH oxidase activity was represented as RLU/s/cell versus Time (min) and AUC value was calculated. At least ten independent experiments were performed for each condition.

### 2.13. Hydrogen Peroxide by Fluorometric Detection

The concentration of H_2_O_2_ produced in the extracellular medium was determined in living CTR, nLES and LES fibroblasts using a commercially available hydrogen peroxide fluorometric detection kit (ADI-907-028, Enzo life sciences AG, Lausen, Switzerland) following manufacturer’s protocol. The Hydrogen Peroxide Fluorometric Detection Kit utilizes a non-fluorescent substrate, 10-Acetyl-3,7- dihydroxyphenoxazine (ADHP), to detect H_2_O_2_. H_2_O_2_ oxidizes ADHP in a one to one ratio to produce a fluorescent product, Resorufin. This oxidation is catalyzed by peroxidase in a homogeneous no-wash assay system. Briefly, 5 × 10^4^ cells per well were plated in black 96-well plate, every sample triplicated. When the cells adhered well to the plate surface (normally, in 4 h), they were washed with pre-heated PBS and left in 50 μL of PBS for 30 min at 37 °C. After that, 50 μL of reaction cocktail containing Horseradish Peroxidase and ADHP were added to the wells. After 10 min incubation at the RT in the darkness, the fluorescence was measured with the fluorometric microplate reader Fluoroskan Ascent (Thermo Electron Corp., Vantaa, Finland). The standard curve was prepared with the H_2_O_2_ ranged in concentration from 0 to 1 μM. The amount of H_2_O_2_ excreted by the fibroblasts was expressed in pM H_2_O_2_ eq./cell. 

### 2.14. Western Blot Analyses

To assess the levels of the proteins of interest, whole-cell lysates were diluted in Laemmli buffer (50 mM TrisHCl, pH 6.9, 10% glycerol, 1.4% 2 mercaptoethanol (ME), 6 M urea, 2% SDS, 0.01% bromphenol blue) and boiled at 96 °C for 5 min. Equal amounts of homogenates (50 µg of protein per line) were separated on 4–12% SDS-PAGE gels (Criterion XT, Bio-Rad Laboratories, Milan, Italy) and transferred to PVDF Hybond membrane (Millipore Corp., Billerica, MA, USA). The membrane was then incubated overnight at 4 °C with a primary antibody (Table 2) diluted in TBS-T buffer (Tris buffered saline (TBS) with Tween-20 (0.1% *w/v*)) with 1% BSA. After washing, the membranes were incubated with peroxidase-conjugated secondary antibodies (Table 2) diluted in TBS-T buffer for 1 h at room temperature. The immune-labelled bands were then detected using a Super-Signal West Dura (Pierce, Rockford, IL, USA) and quantified using ImageJ free software. Results were expressed as the ratio between the densitometries of the protein of interest and loading control GAPDH revealed at the same blot. For this, the blots were stripped with the stripping buffer (100 mM 2-mercaptoethanol, 2% SDS, 62.5 mM Tris, pH 6.8) for 45 min at 50 °C with gentle shaking, washed with TBS-T buffer, re-blocked in TBS-T with 1% BSA for at least 1 h and re-probed with different antibody. The same re-probing protocol was applied also for MAPK molecular pathways activation: p38 and pp38, ERK1/2 and pERK1/2, JNK and pJNK pairs were revealed at the same blots.

### 2.15. Nox4 RNA Interference (RNAi) Assay

CTR, nLES and LES fibroblasts at semi-confluence were washed in pre-heated PBS and left in serum- and antibiotics-free DMEM for 30 min at 37 °C. Further, 50 nM of siNOX4 and Lipofectamin2000 separately diluted in Opti-MEM were mixed at a ratio 2.5/1, respectively, and incubated in a dark at RT for 20 min. Subsequently, the formed si-NOX4-Lipofectamin2000 complexes were added to the cells. In 4–5 h after transfection, the medium was changed with completed DMEM. In 24 h after the transfection (day 1) the cells were used for co-culture experiments. FITC-scRNA was used as a positive control for transfection and was controlled every 24 h following transfection. Transfection level was confirmed to be stable for at least 72 h after transfection and represented at least 80% of transfected cells. NOX4 protein expression was controlled by Western blot in 24 h and in 72 h (not shown) following transfection with siNOX4 RNA. Transfection with siNOX4 RNA suppressed NOX4 by 65–70% (24 h after the transfection) and was effective and didn’t change significantly for at least 72 h after transfection. 

### 2.16. Figure Preparation

Images were assembled in panels using Adobe Photoshop CS (version 10.0) and Adobe Illustrator CS5.1 software provided by the department of Biomedical Experimental and Clinical Sciences, University of Florence (Florence, Italy). When the brightness/contrast were adjusted the exact same procedure was applied to all images of the corresponding panel in order to maintain the eventual differences and allow the images to be comparable to each other. 

The protein bands from the Western blot images were quantified using Image J free software and GraphPad Prism 5 software provided by the department of Biomedical Experimental and Clinical Sciences, University of Florence (Florence, Italy).

### 2.17. Statistical Analysis

The data is expressed as mean ± SD. Comparisons between different groups were performed by one-way analysis of variance followed by Tukey Test with the GraphPad Prism 5 software (San Diego, CA, USA). A *p*-value of <0.05 was accepted as statistically significant.

## 3. Results

### 3.1. Cell Cycle Progression is Altered in LES Fibroblasts

**Figure 2:**
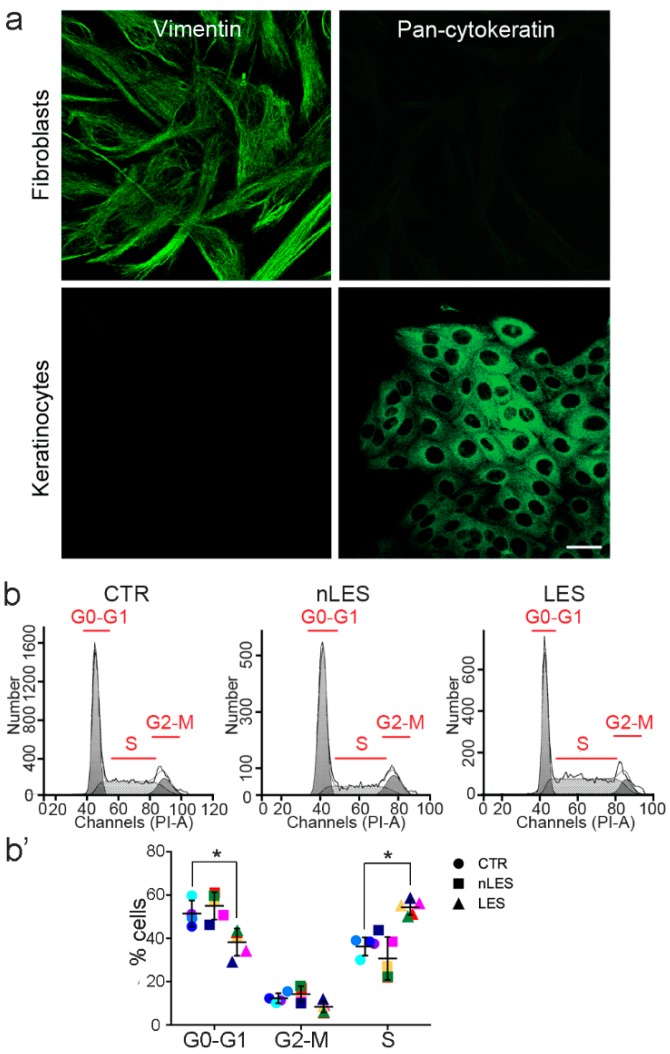
Fibroblasts were obtained from the skin of healthy volunteers (CTR) and from unaffected (nLES) and lesional (LES) skin areas of psoriatic patients; (**a**) Confocal microscopy analysis confirmed the purity of obtained fibroblasts cultures which were positive to anti-vimentin and negative to pan-cytokeratin immunocytochemical staining. (**b**) The cell cycle was analyzed by FACS Analysis in LES, nLES and CTR fibroblasts on their second passage. (**b’**) Cells distribution quantification in G0-G1, G2-M and S phases of the cell cycle. In dot plot graphs, each patient/control is identified by a different color (see Table 3). Values are presented as means ± SD. Each experiment was performed in triplicate. * *p* < 0.05 vs. CTR. Scale bar = 20 μm.

The primary fibroblasts cultures were successfully obtained from CTR, nLES and LES skin samples and immunostained against vimentin and pan-cytokeratin in order to confirm the absence of contamination with keratinocytes (Figure 2a). The cell cycle progression of CTR, nLES and LES fibroblasts was analyzed with PI staining and flow cytometry (Figure 2b). Significantly decreased number of cells entered G0/G1 phase with incrimination of cells entered S phase were shown for LES with respect to CTR and nLES fibroblasts. A non-significant decrease of cell number entered G2/M phase was also noted for LES fibroblasts versus CTR and nLES. No significant difference in cell cycle progression between CTR and nLES fibroblasts was observed (Figure 2b’). The cell cycle was analyzed at passage 2 for psoriatic and control fibroblasts. 

### 3.2. Redox Imbalance in Psoriatic Fibroblasts

**Figure 3:**
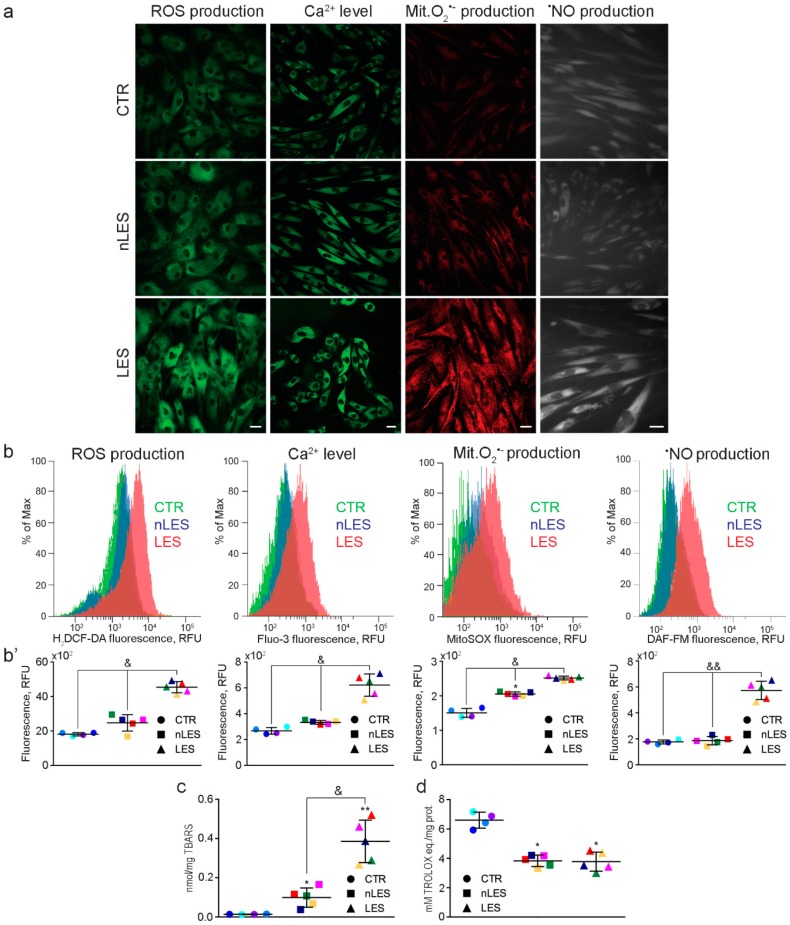
Assessment of redox markers in human psoriatic fibroblasts. For the assessment of the intracellular levels of ROS, Ca^2+^, mitochondrial superoxide anion production and •NO, CTR, nLES and LES fibroblasts were stained with the fluorescent markers H_2_DCFDA, Fluo-3, MitoSOX, and DAF-FM, respectively. (**a**) Confocal microscope analysis, and (**b**,**b’**) FACS analysis revealed signs of oxidative/nitrosative stress in LES fibroblasts. (**c**) Thiobarbituric acid reactive substance (TBARS) assay revealed increased lipid peroxidation in LES with respect to nLES and CTR fibroblasts. (**d**) Decreased total antioxidant capacity measured by oxygen radical antioxidant capacity (ORAC) assay and expressed in Trolox equivalents was evident in psoriatic versus CTR fibroblasts. In dot plot graphs, each patient/control is identified by a different color (see Table 3). Values are presented as means ± SD. Each experiment was performed in triplicate. & *p* < 0.05, && *p* < 0.01. * *p* < 0.05 vs. CTR, ** *p* < 0.01 vs. CTR. Scale bar = 20 μm.

Total intracellular ROS, calcium, mitochondrial O_2_•^−^ and nitric oxide (NO) levels were studied with confocal microscopy (Figure 3a) and flow cytometry (Figure 3b) in living fibroblasts marked with H_2_DCF-DA, Fluo-3, MitoSOX^TM^ and DAF-FM, respectively. Both approaches revealed significantly higher fluorescence levels in LES with respect to CTR and nLES fibroblasts stained with oxidative stress markers. Only mitochondrial O_2_•^−^ production was significantly enhanced in nLES with respect to CTR fibroblasts (Figure 3b’). 

To quantify lipid peroxidation, TBARS were measured in total cell lysates prepared from CTR, nLES and LES fibroblasts (Figure 3c). TBARS concentrations were significantly higher in LES (0.26 ± 0.15 nmol/mL) with respect to nLES (0.12 ± 0.07 nmol/mL) and CTR (0.01 ± 0.001 nmol/mL, *p* < 0.01) and in nLES with respect to CTR (*p* < 0.05). 

Total antioxidant capacity expressed in Trolox equivalents (TE) resulted 6.65 ± 0.31, 3.74 ± 0.60, 3.90 ± 0.66 μmol TE/mg for CTR, nLES and LES, respectively (Figure 3d). Antioxidant capacity was significantly lower in psoriatic with respect to CTR fibroblasts. The obtained data points to a pronounced oxidative stress condition in primary LES and enhanced mitochondrial O_2_•^−^ leakage and decreased antioxidant capacity in nLES with respect to CTR fibroblasts. 

### 3.3. NADPH Oxidase-Mediated Extracellular ROS Production Is Enhanced in LES Fibroblasts

**Figure 4:**
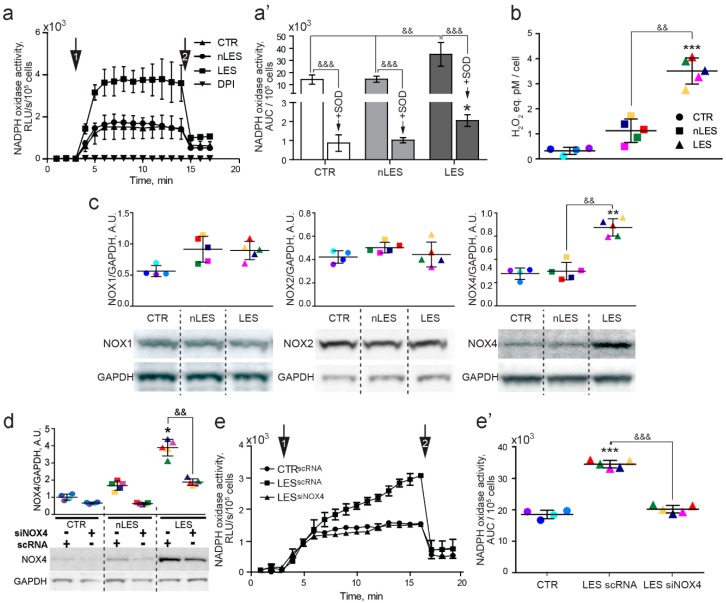
NADPH oxidase activity, expression and intracellular localization in human fibroblasts. NADPH oxidase activity was measured in intact living cells by luminometric assay following NADPH stimulation (arrow 1). Superoxide dismutase (SOD) addition to the reaction mixture (arrow 2) brought to immediate and significant luminescence decrease. (**a**) The diphenylene iodonium chloride (DPI), a potent flavoenzyme inhibitor, abolished NADPH-triggered NADPH oxidase activity in fibroblasts suggesting that extracellular ROS production stimulated by NADPH was NADPH oxidase-dependent. (**a’**) Area Under Curve (AUC) values quantified from the NADPH oxidase activity curves. (**b**) Hydrogen peroxide fluorometric detection in CTR, nLES and LES cell culture medium. (**c**) Western blot analysis of NOX1 (the band detected at 65 kDa, additional band observed at 50 kDa), NOX2 (the band detected at 91 kDa) and NOX4 (the band detected at 67 kDa, additional bands observed at 50 and 20 kDa) expression in fibroblasts. (**d**) Down-regulation of NOX4 with siNOX4 RNAs abolished the differences in NOX4 expression between LES, nLES and CTR fibroblasts. Moreover, (**e**) NADPH oxidase activities were similar in LES^siNOX4^ and CTR^scRNA^ fibroblasts. (**e’**) Quantification analysis of AUC values of NADPH oxidase activity. In dot plot graphs, each patient/control is identified by a different color (see Table 3). Values are presented as means ± SD. Each experiment was performed in triplicate. && *p* < 0.01, &&& *p* < 0.001. * *p* < 0.05 vs. CTR, ** *p* < 0.01 vs. CTR, *** *p* < 0.001 vs. CTR.

Extracellular ROS production was measured in intact living CTR, nLES and LES fibroblasts by luminometric assay. Following stimulation with the substrate (NADPH) an immediate increase in extracellular ROS production was noted for CTR and psoriatic fibroblasts (Figure 4a, arrow 1). NADPH activity curves reached the plateau level of 1484 ± 162, 1417 ± 150 and 3695 ± 330 (RLU/s/105 cells ± SD) for CTR, nLES and LES fibroblasts, respectively. The pre-incubation of fibroblasts with flavoenzyme inhibitor DPI abolished NADPH-stimulated extracellular ROS production in CTR and psoriatic fibroblasts highlighting NADPH oxidase as the source of extracellular ROS production in fibroblasts (Figure 4a). The analysis of AUC values pointed to a significantly higher level of NADPH-triggered ROS production in LES with respect to CTR and nLES fibroblasts (Figure 4a’). The addition of SOD to the reaction mix reduced instantly and significantly the luminescence level by 67.6% ± 15.3%, 64.1% ± 13.5% and 72.3% ± 14.9% for CTR, nLES and LES fibroblasts, respectively (Figure 4a, arrow 2; Figure 4a’). The post-SOD level of luminescence was still significantly increased in LES versus CTR and nLES fibroblasts but not in nLES vs. CTR (Figure 4a’). According to the results of the luminometric assay, firstly, O_2_•^−^ is the main but not the only type of ROS produced by fibroblasts in the extracellular medium and, secondly, LES (vs. CTR and nLES) produce in extracellular space significantly higher amounts of other ROS not derived from superoxide anion.

An independent test estimated the concentration of hydrogen peroxide released by living CTR, nLES and LES fibroblasts in intracellular medium: 0.30 ± 0.16, 1.50 ± 0.90 and 2.86 ± 1.16 pM H_2_O_2_ eq./cell, respectively (Figure 4b). LES resulted to excrete significantly higher amounts of H_2_O_2_ in extracellular medium with respect to CTR and nLES fibroblasts. Instead, although nLES produce higher amounts of H_2_O_2_ with respect to CTR, this difference is not significant (*p* = 0.086).

### 3.4. The Role of NOX4 in ROS Hyper-Production by LES Fibroblasts

To highlight which NADPH oxidase family member is involved in increased extracellular ROS release by LES, the expressions of NOX1, NOX2 and NOX4 were studied in fibroblasts. All three NADPH isoforms, NOX1, NOX2 and NOX4, were expressed in primary human fibroblasts (Figure 4c). No difference in NOX1 and NOX2 expression was found in psoriatic fibroblasts with respect to CTR. Instead, a significantly higher level of NOX4 expression was found in LES versus CTR and nLES fibroblasts. 

In order to investigate whether NOX4 is involved in increased extracellular ROS production in LES fibroblasts, CTR and psoriatic cells were transfected with the mix of three siNOX4 RNAs. The optimal transfection conditions were firstly selected using FITC-marked noncoding RNA (scRNA) and the efficacy of NOX4 knockdown was controlled by western blot analysis. NOX4 expression level was not significantly different between siNOX4-transfected LES, CTR and nLES (Figure 4d). Further, NOX4 knockdown led to a significant decrease (by 43.0 ± 2.3%) in NADPH oxidase activity in LES with respect to LES transfected with scRNA (Figure 4e,e’). Finally, the maximum level of NADPH oxidase activity, as well as AUC, were not different in CTR and siNOX4 LES. Taken together, this data reveals NOX4 as a source of enhanced extracellular ROS production in LES fibroblasts. 

### 3.5. ERK1/2 and p38 Signaling Pathways Activation in Keratinocytes Co-Cultured with Fibroblasts

**Figure 5:**
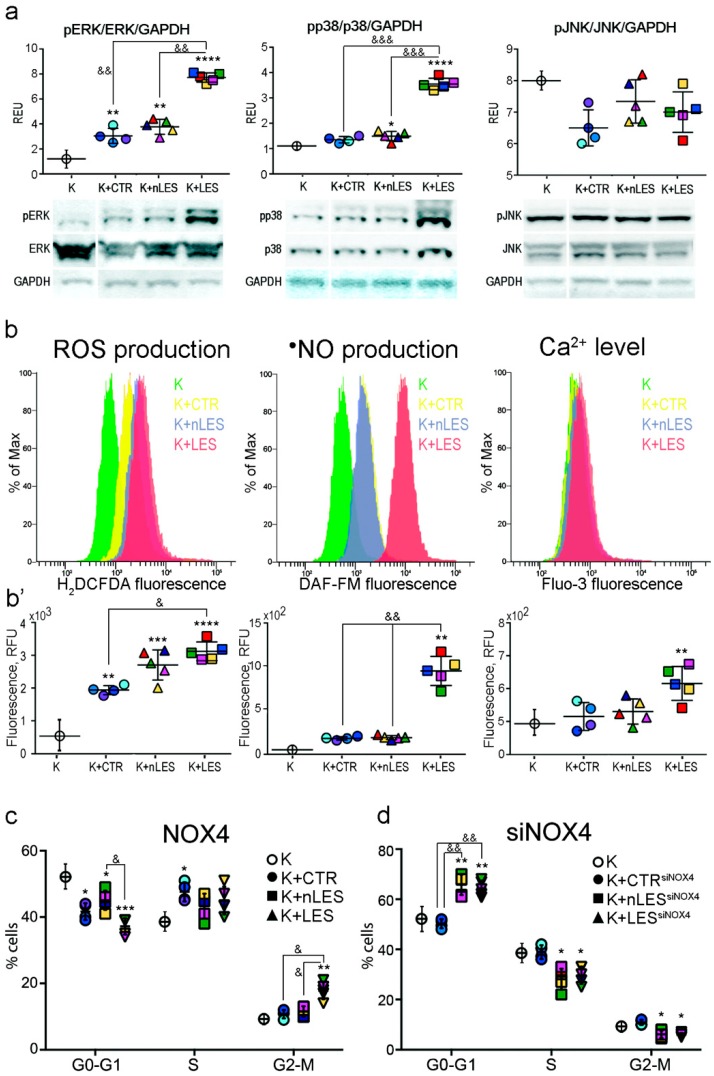
Co-culture with fibroblasts alters MAPK signalling, redox homeostasis and cell cycle in primary human keratinocytes (K). (**a**) Western blot analysis revealed ERK1/2 and p38 pathways activation in K co-cultured with control fibroblasts (K+CTR) and psoriatic non-lesional (K+nLES) and lesional (K+LES) fibroblasts, respectively, with respect to K not in co-culture. Co-culture with fibroblasts didn’t bring to any significant alteration in the JNK pathway in K in our experimental conditions. (**b**) Following the co-culture with fibroblasts, K were stained with H_2_DCFDA, DAF-FM and MitoSOX markers in order to estimate the total intracellular ROS, •NO and Ca^2+^ levels, respectively, by flow cytometry. (**b’**) The quantification of emitted fluorescence assayed by flow cytometer showed that co-culture with fibroblasts significantly increased intracellular ROS production in K. Only co-culture with LES dramatically increased the intracellular •NO and Ca^2+^ content vs. K. (**c**) Co-incubation with fibroblasts and, especially, with LES, altered the distribution of K in G0-G1, S and G2-M phases of cell cycle measured by FACS Analysis. (**d**) Transfection of fibroblasts with siNOX4 RNAs induced the accumulation of K in G0-G1 phase of the cell cycle. In dot plot graphs, each patient/control is identified by a different color (see Table 3). Values are presented as means ± SD. Each experiment was performed in triplicate. & *p* < 0.05, && *p* < 0.01, &&& *p* < 0.001; * *p* < 0.05 vs. K, ** *p* < 0.01 vs. K, *** *p* < 0.001 vs. K, **** *p* < 0.0001 vs. K.

First of all, the activation of three main MAPK molecular pathways (ERK, p38 and JNK) was analyzed by western blot in Keratinocytes (K) co-cultured with fibroblasts (Figure 5a). A significantly increased ERK phosphorylation (pERK) was observed in K co-cultured with CTR, nLES and LES fibroblasts with respect to K non-co-incubated with fibroblasts. Moreover, co-culture with LES brought to a significantly higher level of ERK phosphorylation with respect to CTR and nLES. Furthermore, a strong increase in phosphorylated p38 (p-p38) expression was noted in K co-incubated with LES; while in K co-incubated with nLES the increase in p-p38 was less evident but still significant. Finally, no activation of JNK molecular pathways was observed in K co-cultured with fibroblasts in our experimental conditions. The activation of pro-mitotic (ERK) and stress-sensitive (p38) molecular pathways correlated with the results discussed below.

### 3.6. LES Fibroblasts Alter Keratinocytes Redox Balance in Co-Culture

Following co-culture with fibroblasts, K were stained with H_2_DCFDA, DAF-FM and Fluo-3 fluorescent markers in order to study the levels of intracellular ROS, •NO and Ca^2+^, respectively (Figure 5b,b’). The co-incubation with fibroblasts brought to a significant increase in intracellular ROS levels in K. Importantly, the intracellular ROS production was significantly higher in K co-incubated with LES vs. K co-incubated with CTR. Interestingly, only in K co-cultured with LES the significant increase of intracellular •NO and Ca^2+^ levels were observed.

### 3.7. Co-Culture with Fibroblasts Affects Cell Cycle Progression in Keratinocytes

Finally, the K co-incubated with the fibroblasts were stained with propidium iodide to study cellular distribution in the phases of the cell cycle by flow cytometry (Figure 5c). Significant alterations in cell cycle progression of K co-cultured with the fibroblasts with respect to K non in co-culture were revealed. The co-incubation with CTR, nLES and LES fibroblasts brought a significant decrease in G1/G0 phase population (by 18.2% ± 1.8%, 15.3% ± 2.9% and 30.8% ± 5.4% vs. CtrKER, respectively) although the differences were more pronounced in K co-incubated with LES. A slight increase in S phase population was also noted in K co-incubated with fibroblasts: by 21.6% ± 3.0%, 14.3% ± 3.9% and 15.7% ± 3.4% in KER co-incubated with CTR, nLES and LES fibroblasts, respectively, vs. K not in co-culture. Finally, only co-incubation with LES fibroblasts brought to a significant increase of G2 phase population (by 117.6% ± 58.2%) in K. Although all types of fibroblasts accelerated the K cell cycle progression, this effect was significantly more pronounced in case of K co-culture with LES fibroblasts. 

### 3.8. ROS Over-Produced by NOX4 in LES Fibroblasts are Mitogens for Keratinocytes in Co-Culture

**Figure 6:**
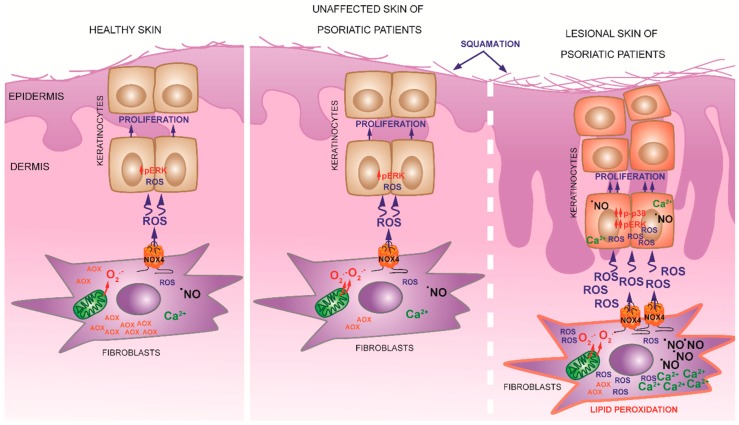
Schematic representation of the proposed mechanism of NOX4 contribution in psoriatic plaque formation. Healthy primary fibroblasts, as well as fibroblasts obtained from visibly unaffected skin of psoriatic patients, activate ERK1/2, increase intracellular total ROS level and accelerate cell cycle progression in healthy primary keratinocytes in co-culture through the redox signalling mediated by NOX4. Fibroblasts obtained from unaffected and lesional skin of psoriatic patients differ from healthy fibroblasts by elevated mitochondrial superoxide anion production and decreased total antioxidant capacity. Moreover, lesional fibroblasts display significantly higher levels of intracellular •NO, Ca^2+^ and lipid peroxidation as well as elevated NOX4 activity and extracellular ROS production. Lesional fibroblasts induce significantly higher levels of intracellular ROS and ERK activation in healthy keratinocytes with respect to healthy and unaffected psoriatic fibroblasts. Moreover, co-culture with lesional fibroblasts induced •NO and Ca^2+^ accumulation in keratinocytes and significant activation of p38 molecular pathway. The result of these metabolic alterations is keratinocytes hyper-proliferation. Hence, dermal fibroblasts may trigger keratinocytes proliferation through NOX4-mediated redox signalling.

In order to study the role of ROS overproduced by LES in K cell cycle, the CTR, nLES and LES fibroblasts were transfected with siNOX4-RNA and co-cultivated with K for 48 h. NOX4 knockdown abolished the observed mitogen effect of fibroblasts versus K (Figure 5d). Moreover, a significant increase in the amount of K stocked in G0/G1 phase in concomitance with significantly decreased S- and G2/M-phase keratinocytes were observed following co-incubation with siNOX4-nLES and -LES fibroblasts. Importantly, no significant difference in cell cycle progression in K not in co-culture and siNOX4-CTR-co-incubated keratinocytes were noted. Taken together these results point out that ROS over-produced by fibroblasts play a mitogenic role for keratinocytes in co-culture (Figure 6).

## 4. Discussion

In the present study, we describe in detail the redox features of fibroblasts obtained from lesional (LES) and non-lesional (nLES) skin of psoriatic patients in comparison to control fibroblasts (CTR). nLES fibroblasts did not manifest any significant alteration in intracellular Ca^2+^, •NO and ROS which, instead, were significantly increased in LES fibroblasts compared to CTR. However, a significant increase in mitochondrial superoxide production was correlated with the increased lipid peroxidation and decreased total antioxidant capacity in nLES with respect to CTR fibroblasts. Although in-depth studies on lipid peroxidation should be conducted, our preliminary data indicate the presence of a redox shift towards higher oxidation in nLES vs. CTR in visually unaffected skin of psoriatic patients. This could explain the higher sensitivity of the patients’ skin to develop the psoriatic plaques under exposure to intrinsic and extrinsic factors. 

The acute imbalance of intracellular ROS, Ca^2+^ and •NO observed in LES fibroblasts confirms that diverse cellular signalling events are regulated by the concomitant increase in these transients [13]. NOX4, which was found to be over-expressed in LES fibroblasts, was shown to be an upstream regulator of nitric oxide synthase (NOS) [14]. Importantly, increased •NO synthesis can display a role in psoriasis pathogenesis as it may enhance the release and actions of calcitonin gene-related peptide and substance P, which can induce the production of adhesion molecules, keratinocyte hyper-proliferation, mast cell degranulation, vasodilatation, and chemotaxis of neutrophils. Some authors also reported that •NO stimulates epithelial cells to release chemokines and growth mediators which appear to be important for keratinocyte proliferation and angiogenesis [15]. Our findings indicate that increased NOX4 expression parallels the significantly enhanced NADPH oxidase-dependent extracellular ROS production in LES fibroblasts. Indeed, NOX4 is the only member of NADPH oxidase family that is constitutively active and regulated on its expression level [16]. The extracellular production of H_2_O_2_ by LES was confirmed in this study with two independent approaches. Importantly, enhanced mitochondrial activity in LES did not contribute to extracellular ROS over-production: ROS production was completely abolished by NADPH oxidase inhibitor diphenylene iodonium and was not different between CTR and nLES fibroblasts, where mitochondrial O_2_•^−^ production was significantly increased. These findings demonstrate that fibroblasts obtained from the area of psoriatic plaque are characterized by altered redox balance and by a significantly enhanced NOX4 expression and activity that persists through several passages in vitro confirming the involvement of endogenous mechanisms. In our study, conducted on five psoriatic and on four control samples, we didn’t take into consideration the possible gender-associated differences in intracellular ROS production and in NADPH oxidases expression. Indeed, according to recent data obtained on animal models, although no uniform consensus reached, there may be sex-dependent differences in NOX isoforms expression [17]. However, the present study was conducted on isolated cell cultures not exposed to gender-related blood factors, such as estrogen, and here we discuss the macro-effects exerted by fibroblasts on keratinocytes redox regulation. Thus, we speculate that for the scope of this study and in accordance with the obtained results, the impact of the patients’ sex can be neglected. Certainly, when passing to the animal model, gender will be taken into consideration.

The complex distribution of NOX4 in the human fibroblasts evidenced by the confocal microscopy and immunocytochemical staining confirms the role of NOX4 in redox signalling in physiological and pathological conditions. Indeed, other authors have shown on A549 and human heart tissue that the alternative splicing of NOX4 brings to the presence of several NOX4 isoforms that differ in intracellular localization [18,19]. The study of splicing isoforms in human skin fibroblasts and keratinocytes as well as the expression of other two members of NADPH oxidase family releasing H_2_O_2_ as the main product, DUOX1 and DUOX2, may be the purpose of further studies.

In order to understand if the increased ROS production by fibroblasts in the extracellular medium could affect keratinocytes in co-culture, we established an easy and low-cost model of contactless co-culture of keratinocytes with CTR, nLES or LES fibroblasts. Interestingly, all types of fibroblasts significantly affected keratinocytes. In particular, we observed in keratinocytes increased ERK1/2 phosphorylation, higher intracellular ROS and cell cycle acceleration. These findings suggest that fibroblasts-derived factor(s) are able to activate the mitogenic ERK pathway resulting in increased keratinocytes proliferation. Indeed, existing data highlights that the canonical Ras–Raf–MEK–ERK MAPK pathway can activate cyclin D transcription and, thus, accelerate cell cycle progression [20]. Importantly, the ERK pathway can be directly activated by ROS [21]. 

A significantly higher ERK activation, intracellular ROS accumulation and cell cycle progression rate were evident in keratinocytes co-incubated with LES fibroblasts. The significant increase in p38 phosphorylation only in keratinocytes co-cultured with LES suggests that p38 activation may be dependent on ROS concentration. Indeed, p38 and JNK pathways are stress-sensitive pathways activated by multiple stress stimuli including H_2_O_2_ [22]. A different time-dependent activation of the JNK pathway by H_2_O_2_ was shown for distinct human cell types [23]; this can explain the absence of pJNK activation in keratinocytes co-cultured with the fibroblasts in our conditions. Co-culture with LES fibroblasts significantly increased •NO and Ca^2+^ levels in keratinocytes confirming again the tight interference between ROS and these signalling species. 

It has been already proposed that fibroblasts could produce pro-proliferative factors for keratinocytes in psoriatic lesions. Among others, the attention was given to cytokines. Debets and co-authors [24] have shown that psoriatic fibroblasts release increased amounts of IL-6 with respect to control fibroblasts. It was also demonstrated that IL-6 along with some other cytokines (for instance, IL-4 and IL-8) and growth factors mediate epidermal hyperplasia [25]. At the same time, ROS can stimulate proliferation: Kim and co-authors have shown that the treatment of keratinocytes with H_2_O_2_ (100 μM) or superoxide anion (generated by 1 mM xanthine and 1 mU/mL xanthine oxidase) increased proliferation rate by approximately 50% [26]. Impressively, according to our data, NOX4 silencing not only abolished the mitogenic effect of fibroblasts on keratinocytes but also resulted in higher accumulation of cells in G0/G1 phase in case of co-culture with LES fibroblasts. Thus, our data demonstrate that NOX4-mediated redox signalling has a central role in the fibroblast/keratinocyte cross-talk.

Taken together, the findings here reported indicate a marked redox imbalance in LES fibroblasts associated with increased intracellular oxidative stress markers and excessive extracellular NOX4-derived ROS production. Future studies have to elucidate the mechanism through which NOX4 remains hyper-activated in vitro for at least seven passages of LES fibroblasts. The search in PheGenI and EMBL-EBI databases didn’t reveal any NOX4 polymorphism associated with psoriasis. Thus, the dermis is able to generate and maintain the pathologic condition associated with the NOX4 hyper-activation in the absence of external stimuli highlighting the possible role of epigenetic mechanisms. Indeed, it was shown that the NOX4 mRNA expression is finely regulated by epigenetic mechanisms, involving microRNA-dependent posttranscriptional repression [27]. Studies are in progress to reveal novel molecular mechanisms of NOX4 regulation and paracrine redox signalling.

## 5. Conclusions

For the first time, the role of dermal fibroblasts in the redox-mediated modulation of keratinocyte metabolism was demonstrated (Figure 6). Fibroblasts to keratinocytes redox relation should be taken into consideration for the setting up of new therapeutic approaches for inflammatory skin diseases as well as for preventive cosmetological skin treatments. Collectively, more attention should be dedicated to the dermis in preventive and curative treatments. Future studies dealing with the molecular mechanisms of NOX4 regulation in skin fibroblasts are needed in order to set up novel approaches to modulate skin physiology. 

## Figures and Tables

**Table 1:** Reagents used in the study.

Reagent	Abbreviation	Source
2,2′-azobis-2-amidinopropanedihydrochloride	AAPH	1
Aprotinin		1
Catalase	CAT	1
Diphenylene iodonium	DPI	1
Dispase II		1
Dulbecco’s Modified Eagle’s Medium	DMEM	1
Dulbecco’s Phosphate Buffered Saline	PBS	1
Fluorescein		1
Leupeptin		1
l-glutamine		1
Lucigenin		1
Penicillin-Streptomycin solution		1
Antibiotic-Antimycotic Solution		1
Phenylmethylsulfonyl fluoride	PMSF	1
Roswell Park Memorial Institute-1640 medium	RPMI	1
Superoxide dismutase	SOD	1
6-hydroxy-2,5,7,8-tetramethylchroman-2-carboxylic acid	Trolox	1
β-Nicotinamide adenine dinucleotide 2′-phosphate	NADPH	1
2′,7′-dichlorodihydrofluorescein diacetate (ROS indicator)	H_2_DCFDA	2
4-Amino-5-Methylamino-2′,7′-Difluorofluorescein Diacetate (nitric oxide indicator)	DAF-FM DA	2
Fluo-3 AM (calcium indicator)		2
Red Mitochondrial Superoxide Indicator	MitoSOX™	2
Lipofectamine^TM^ 2000 Transfection reagent		3
Gibco^TM^ Opti-Minimal Essential Medium	MEM	3
Fetal bovine serum	FBS	3
Control siRNA (Fluorescent Conjugate-A, sc-36869)		4
(h) NOX4 siRNA (sc-41586)		4
Keratinocyte Growth Medium^TM^	KGM^TM^	5
KGM^TM^-2 SingleQuotsTM (Supplements and Growth Factors)		5
DAPI		
BSA	BSA	1
Trypsin-EDTA solution		1

1 Sigma-Aldrich Italy S.r.l., Milan, Italy; 2 Thermo Fisher Scientific Inc., Monza, Italy; 3 Invitrogen, Grand Island, NY, USA; 4 Santa Cruz Biotechnology, Inc., Santa Cruz, CA, USA; 5 Lonza Bioscience, Basel, Switzerland.

**Table 2:** Antibodies used in the study.

**Primary Antibody**	**Host Species**	**Code**	**Dilutions**	**Source**
anti-NOX4	rabbit polyclonal	ab109225	WB: 1:1000	2
anti-gp91-phox (anti-NOX2)	goat polyclonal	C-15 sc-5827	WB: 1:500	1
anti-NOX1	goat polyclonal	ab121009	WB: 1:1000	2
anti-ERK1/2	rabbit polyclone	sc-101761	WB: 1:500	1
anti-p-ERK	mouse monoclonal	sc-7383	WB: 1:500	1
anti-p38	mouse monoclonal	sc-7972	WB: 1:500	1
anti-p-p38	mouse monoclonal	sc-166182	WB: 1:500	1
anti-pJNK	mouse monoclonal	sc-6254	WB: 1:500	1
anti-GAPDH	rabbit polyclonal	sc-25778	WB: 1:500	1
anti-vimentin	mouse monoclonal	sc-6260	ICC: 1:1000	1
anti-pan-Cytokeratin	mouse monoclonal	sc-8018	ICC: 1:1000	1
**Secondary Antibody**	**Host Species**	**Code**	**Dilutions**	**Source**
anti-rabbit IgG-FITC	goat	sc-2012	ICC: 1:1000	1
anti-goat IgG-HRP	donkey	sc-2020	WB: 1:5000	1
anti-mouse IgG-HRP	goat	sc-2005	WB: 1:5000	1
anti-rabbit IgG-HRP	goat	1858415	WB: 1:8000	3

1 Santa Cruz Biotechnology, Inc., Santa Cruz, CA, USA; 2 Abcam plc., Cambridge, UK; 3 Pierce from Thermo Fisher Scientific Inc., Monza, Italy.

**Table 3:** Demographic and clinical data of patients and healthy controls involved in the study. Different colours were assigned to each psoriatic patient and control in order to trace the corresponding primary fibroblasts in each experiment. Colors of each patient/control were maintained throughout the manuscript.

Patients	Age	BMI	Duration of Disease (Years)	PASI	Biopsy Region	Healthy Volunteers (CTR)	Age	BMI	Biopsy Region
**PSO.M1**	33	25	8	13	Arm	**CTR.M1**	32	25	Abdomen
**PSO.M2**	41	24	12	13	Arm	**CTR.M2**	35	25	Abdomen
**PSO.M3**	29	25	10	12	Abdomen	**CTR.F1** *	39	23	Abdomen
**PSO.F1**	32	24	12	12	Abdomen	**CTR.F2** *	42	24	Arm
**PSO.F2**	37	25	9	12	Arm				
Mean ± SD	34.4 ± 4.6	24.6 ± 0.5	10.2 ± 1.7	12.4 ± 0.5		Mean ± SD	37.0 ± 4.3	24.2 ± 0.9	

* pre-menopausal.

## References

[1] Lisse T.S., King B.L., Riegerb S. (2016). Comparative transcriptomic profiling of hydrogen peroxide signaling networks in zebrafish and human keratinocytes: Implications toward conservation, migration and wound healing. Sci. Rep..

[2] Stone J.R., Yang S. (2006). Hydrogen peroxide: A signaling messenger. Antioxid. Redox Signal..

[3] Meyer T., Wirtz P.H. (2018). Mechanisms of Mitochondrial Redox Signaling in Psychosocial Stress-Responsive Systems: New Insights into an Old Story. Antioxid. Redox Signal..

[4] Zorov D.B., Juhaszova M., Sollott S.J. (2014). Mitochondrial Reactive Oxygen Species (ROS) and ROS-Induced ROS Release. Physiol. Rev..

[5] Nisimoto Y., Ogawa H., Kadokawa Y., Qiao S. (2018). NADPH oxidase 4 function as a hydrogen peroxide sensor. J. Biochem..

[6] Barygina V., Becatti M., Lotti T., Taddei N., Fiorillo C. (2016). Low dose cytokines reduce oxidative stress in primary lesional fibroblasts obtained from psoriatic patients. J. Dermatol. Sci..

[7] Er-raki A., Charveron M., Bonafe J.L. (1993). Increased superoxide anion production in dermal fibroblasts of psoriatic patients. Skin Pharmacol..

[8] Dimon-Gadal S., Gerbaud P., Thérond P., Guibourdenche J., Anderson W.B., Evain-Brion D., Raynaud F. (2000). Increased oxidative damage to fibroblasts in skin with and without lesions in psoriasis. J. Investig. Dermatol..

[9] Becatti M., Barygina V., Mannucci A., Emmi G., Prisco D., Lotti T., Fiorillo C., Taddei N. (2018). Sirt1 Protects against Oxidative Stress-Induced Apoptosis in Fibroblasts from Psoriatic Patients: A New Insight into the Pathogenetic Mechanisms of Psoriasis. Int. J. Mol. Sci..

[10] Becatti M., Barygina V., Emmi G., Silvestri E., Taddei N., Lotti T., Fiorillo C. (2016). SIRT1 activity is decreased in lesional psoriatic skin. Intern. Emerg. Med..

[11] Barygina V., Becatti M., Mannucci A., Taddei N., Tirant M., Hercogovấ J., França K., Fioranelli M., Roccia M.G., Tchernev G. (2016). Rapid communication: A vegetable oil extract restores redox status in fibroblasts from psoriatic patients. J. Biol. Regul. Homeost. Agents.

[12] Bradford M.M. (1976). A rapid and sensitive method for the quantitation of microgram quantities of protein utilizing the principle of protein–dye binding. Anal. Biochem..

[13] Hempel N., Trebak M. (2017). Crosstalk between calcium and reactive oxygen species signaling in cancer. Cell Calcium.

[14] Lee D.Y., Wauquier F., Eid A.A., Roman L.J., Ghosh-Choudhury G., Khazim K., Block K., Gorin Y. (2013). Nox4 NADPH oxidase mediates peroxynitrite-dependent uncoupling of endothelial nitric-oxide synthase and fibronectin expression in response to angiotensin II: Role of mitochondrial reactive oxygen species. J. Biol. Chem..

[15] Cals-Grierson M.M., Ormerod A.D. (2004). Nitric oxide function in the skin. Nitric Oxide.

[16] Breitenbach M., Rinnerthaler M., Weber M., Breitenbach-Koller H., Karl T., Cullen P., Basu S., Haskova D., Hasek J. (2018). The defense and signaling role of NADPH oxidases in eukaryotic cells: Review. Wien. Med. Wochenschr..

[17] Kander M.C., Cui Y., Liu Z. (2017). Gender difference in oxidative stress: A new look at the mechanisms for cardiovascular diseases. J. Cell. Mol. Med..

[18] Goyal P., Weissmann N., Rose F., Grimminger F., Schäfers H.J., Seeger W., Hänze J. (2005). Identification of novel Nox4 splice variants with impact on ROS levels in A549 cells. Biochem. Biophys. Res. Commun..

[19] Varga Z.V., Pipicz M., Baán J.A., Baranyai T., Koncsos G., Leszek P., Kuśmierczyk M., Sánchez-Cabo F., García-Pavía P., Brenner G.J. (2017). Alternative Splicing of NOX4 in the Failing Human Heart. Front. Physiol..

[20] Duronio R.J., Xiong Y. (2013). Signaling pathways that control cell proliferation. Cold Spring Harb. Perspect. Biol..

[21] León-Buitimea A., Rodríguez-Fragoso L., Lauer F.T., Bowles H., Thompson T.A., Burchiel S.W. (2012). Ethanol-induced oxidative stress is associated with EGF receptor phosphorylation in MCF-10A cells overexpressing CYP2E1. Toxicol. Lett..

[22] Liu Y., Wang N., Zhang S., Liang Q. (2018). Autophagy protects bone marrow mesenchymal stem cells from palmitate induced apoptosis through the ROS JNK/p38 MAPK signaling pathways. Mol. Med. Rep..

[23] Mantzaris M.D., Bellou S., Skiada V., Kitsati N., Fotsis T., Galaris D. (2016). Intracellular labile iron determines H2O2-induced apoptotic signaling via sustained activation of ASK1/JNK-p38 axis. Free Radic. Biol. Med..

[24] Debets R., Hegmans J.P., Deleuran M., Hooft S., Benner R., Prens E.P. (1996). Expression of cytokines and their receptors by psoriatic fibroblast. I. Altered IL-6 synthesis. Cytokine.

[25] Gallucci R.M., Sloan D.K., Heck J.M., Murray A.R., O’Dell S.J. (2004). Interleukin 6 indirectly induces keratinocyte migration. J. Investig. Dermatol.

[26] Kim B.Y., Han M.J., Chung A.S. (2001). Effects of reactive oxygen species on proliferation of Chinese hamster lung fibroblast (V79) cells. Free Radic. Biol. Med..

[27] Varga Z.V., Kupai K., Szucs G., Gaspar R., Paloczi J., Farago N., Zvara A., Puskás L.G., Rázga Z., Tiszlavicz L. (2013). MicroRNA-25-dependent up-regulation of NADPH oxidase 4 (NOX4) mediates hypercholesterolemia-induced oxidative/nitrative stress and subsequent dysfunction in the heart. J. Mol. Cell. Cardiol..

